# Reovirus Low-Density Particles Package Cellular RNA

**DOI:** 10.3390/v13061096

**Published:** 2021-06-08

**Authors:** Timothy W. Thoner, Xiang Ye, John Karijolich, Kristen M. Ogden

**Affiliations:** 1 Department of Pathology, Microbiology, and Immunology, Vanderbilt University Medical Center, Nashville, TN 37232, USA; timothy.w.thoner@vanderbilt.edu (T.W.T.J.); xiang.ye@vumc.org (X.Y.); john.karijolich@vumc.org (J.K.); 2 Department of Pediatrics, Vanderbilt University Medical Center, Nashville, TN 37232, USA

**Keywords:** packaging, segmented, dsRNA, *Reoviridae*, reovirus, top component

## Abstract

Packaging of segmented, double-stranded RNA viral genomes requires coordination of viral proteins and RNA segments. For mammalian orthoreovirus (reovirus), evidence suggests either all ten or zero viral RNA segments are simultaneously packaged in a highly coordinated process hypothesized to exclude host RNA. Accordingly, reovirus generates genome-containing virions and “genomeless” top component particles. Whether reovirus virions or top component particles package host RNA is unknown. To gain insight into reovirus packaging potential and mechanisms, we employed next-generation RNA-sequencing to define the RNA content of enriched reovirus particles. Reovirus virions exclusively packaged viral double-stranded RNA. In contrast, reovirus top component particles contained similar proportions but reduced amounts of viral double-stranded RNA and were selectively enriched for numerous host RNA species, especially short, non-polyadenylated transcripts. Host RNA selection was not dependent on RNA abundance in the cell, and specifically enriched host RNAs varied for two reovirus strains and were not selected solely by the viral RNA polymerase. Collectively, these findings indicate that genome packaging into reovirus virions is exquisitely selective, while incorporation of host RNAs into top component particles is differentially selective and may contribute to or result from inefficient viral RNA packaging.

## 1. Introduction

Packaging of segmented, double-stranded RNA (dsRNA) genomes by eukaryotic viruses is an incompletely understood process that requires the coordination of up to 12 viral RNA species as well as structural and non-structural proteins [1,2,3,4]. Two major packaging models have been proposed: (i) a concerted model in which *trans*-interactions between plus-strand (+) RNA species of each segment promote formation of a packageable supramolecular complex that is subsequently encapsidated by viral structural proteins, and (ii) a core-filling model wherein each segment is individually packaged into a preformed core particle [1,2]. Viral nonstructural proteins may act as chaperones and facilitate RNA–RNA interactions that aid in selective RNA packaging [5,6]. Following viral +RNA packaging, minus-strand RNA is synthesized to form dsRNA genome segments, which are present in particles in equimolar proportions. Evidence suggests that the *Reoviridae* family of viruses with segmented, dsRNA genomes employ concerted packaging. However, many questions about how these viruses package their multi-partite genomes remain unanswered.

Mammalian orthoreovirus (reovirus) is a useful model for studies of RNA packaging by viruses belonging to the *Reoviridae* family. Reovirus has a broad host range, has been implicated in the loss of oral tolerance to gluten associated with celiac disease, and is under investigation for its oncolytic therapeutic potential [7,8]. Prototype strains that represent two of the major reovirus serotypes include Type 1 Lang (T1L) and Type 3 Dearing (T3D) [8]. These viruses differ in cell tropism, induction of cell responses, including innate immune signaling and cell death pathways, and pathogenesis in mouse models of disease. Robust reverse genetics systems permit genetic manipulation of T1L and T3D reoviruses [9,10]. Reovirus particles exhibit icosahedral symmetry and are organized into two concentric capsid layers that encapsidate a genome composed of ten dsRNA genome segments, three large (L1–L3), three medium (M1–M3), and four small (S1–S4), that are present in equimolar amounts in purified particles [11]. Following host cell entry and escape from the endosome, transcriptionally active reovirus core particles release viral +RNA into the cytoplasm, where newly translated viral structural and non-structural proteins associate with one another and the host cell cytoskeleton to establish inclusions, also known as virus factories, which serve as sites of progeny virus assembly [8]. Reovirus +RNAs are capped, non-polyadenylated, and typically contain a single open reading frame (ORF) flanked by 5′ and 3′ untranslated regions (UTRs). Within virus factories, assembling reovirus cores package viral +RNAs and an estimated 12 copies of the viral RNA-dependent RNA polymerase (RdRp) and synthesize minus-strand RNA to form the dsRNA genome [8,12]. Newly assembled core particles undergo secondary transcription, synthesizing additional viral +RNA within virus factories [13,14].

While studies of reovirus RNAs, proteins, and particles have yielded insights into packaging, many facets of this complex problem remain incompletely understood. Reovirus RNA packaging signals are thought to reside in the 5′ and 3′ UTRs and extend into the adjacent ORF, with a sequence element in the 5′ end and structural elements in the 3′ end potentially contributing to packaging [9,15,16,17,18,19,20]. Rotavirus non-structural protein NSP2 binds viral +RNA, influences its structure, and is predicted to help nucleate virus assembly [1,21]. Reovirus non-structural proteins µNS and σNS, the latter of which is a predicted rotavirus NSP2 homolog, associate with reovirus +RNA and are components of assembling reovirus particles [4,22]. The reovirus RdRp λ3 is thought to associate with +RNA at each of the 5-fold icosahedral vertices, interacting preferentially with molecules containing G or U in the penultimate position [23,24,25]. Thus, non-structural proteins and λ3 may play important roles in packaging the reovirus genome. Finally, reovirus primarily generates two species of particles that can be separated based on differences in density [26]. Higher-density virions appear “full” of RNA by negative-stain electron microscopy (EM) analysis and contain the complete viral genome. Lower density top component (TC) particles have indistinguishable protein composition to virions but appear “empty” of RNA by negative-stain EM and cryo-EM [26,27,28]. Together, these characteristics suggest reovirus packaging is a highly regulated process resulting in encapsidation of either a complete set of viral genome segments or no segments at all [1,2]. However, TC particles are reported to retain a level of infectivity, albeit a low level, even after multiple sequential rounds of purification, which is inconsistent with the complete lack of packaged viral RNA [28].

In addition to packaging the viral genome, many viruses package host cell RNA species. Host RNA packaging is especially common for retroviruses, including Rous sarcoma virus, Moloney murine leukemia virus, and human immunodeficiency virus, as well as for some bipartite and tripartite single-stranded RNA viruses such as brome mosaic virus and Flock House virus (FHV) [29,30,31,32,33]. There is tremendous variability in the amount of host RNA packaged by different viruses, with host RNA constituting up to 30% of total RNA in retrovirus virions but only about 1% in FHV virions [32,34,35]. Many different types of host RNA can be packaged by viruses, including non-coding RNA (ncRNA), messenger RNA (mRNA), and endogenous retroelement RNAs [35]. FHV virus-like particles, which are formed from expressed viral proteins in the absence of viral nucleic acids, package significantly more host RNA than do virions [32]. The presence of equimolar ratios of packaged segments and an apparent “all-or-none” packaging strategy suggest that reovirus assortment and packaging are exquisitely specific and that host RNA is unlikely to be packaged. Studies of bluetongue virus (BTV) suggest that the smallest genome segments form *trans*-segment interactions that nucleate assembly of RNA complexes containing a full complement of genomic segments [36,37,38]. There also is evidence for stable, sequence-specific interactions between rotavirus +RNAs [21]. These studies further underscore the orderly nature of RNA packaging by viruses in the *Reoviridae* family. However, the detection of rotaviruses with segments containing duplications that have arisen following natural infection or laboratory passage and the recovery of recombinant rotaviruses engineered to contain duplicated or exogenous sequences up to 900 bp in length suggest that there is at least some available space for packaging of additional RNA [39,40,41,42,43]. Reovirus TC particles presumably have even more space available inside the particle. However, whether viruses in the *Reoviridae* family package host RNA is currently unknown.

Through the current study, we sought to gain insight into RNA packaging by viruses in the *Reoviridae* family using reovirus as a model system. Reovirus virions and TC particles served as tools to elucidate reovirus packaging potential. We enriched for reovirus virions and TC particles and defined their RNA content using next-generation RNA-sequencing (NGS). As anticipated, reovirus virions almost exclusively packaged viral dsRNA, with enrichment of very few host-derived RNAs. In contrast, reovirus TC particles were selectively enriched for numerous host RNA species, which constituted a substantial percentage of overall RNA content. Host RNA selection by TC particles was not dependent on RNA abundance in the cell, and specifically enriched host RNAs varied for two reovirus strains independent of the viral RdRp. While the precise features of host RNA that facilitate packaging into TC particles remain to be elucidated, these findings suggest that genome packaging into reovirus virions is exquisitely selective, while RNA packaging into reovirus TC particles is more promiscuous than that of virions, yet selective nonetheless.

## 2. Materials and Methods

### 2.1. Cell Culture

L929 murine fibroblasts (L cells) were maintained in suspension in glass bottles containing a magnetic stir bar or as monolayers in flasks in Joklik’s minimum essential medium (JMEM; US Biological, Salem, MA, USA). Baby hamster kidney cells expressing bacteriophage T7 RNA polymerase under the control of a cytomegalovirus promoter (BHK-T7; [42] were maintained in Dulbecco’s minimum essential medium (DMEM; Corning, Corning, NY, USA) and were treated with 1 mg/mL geneticin (Gibco, Waltham, MA, USA) every other passage. All media were supplemented with 5% fetal bovine serum (FBS; Gibco), 2 mM L-glutamine, 100 U/mL penicillin, 100 µg/mL streptomycin (Corning), and 25 ng/mL amphotericin B.

### 2.2. Viruses

Recombinant strain (rs) T1L and rsT3D^I^T1L1 were generated by reverse genetics [9,10]. rsT3D^I^T1L1 is a T3D reovirus into which a T249I mutation has been engineered in the attachment protein that renders it resistant to proteolytic cleavage, and the λ3-encoding T3D L1 gene has been replaced with that of T1L [9]. BHK-T7 cells at ~50% confluency in 6-well plates were transfected with 0.8 µg of each plasmid encoding the ten T1L or T3D^I^T1L1 genome segments using TransIT LT-1 Reagent (Mirus Bio LLC, Madison, WI, USA). Transfected cells were cultured for 5 days or until the first signs of cytopathic effects before freezing at −80 °C and thawing at room temperature twice to release virus into supernatant. Virus was then amplified in L cells for two passages. RNA was extracted from virus stocks, and L1 and S1 identities were verified by Sanger sequencing. Virus titer was determined by standard plaque assay [44].

### 2.3. Reovirus Particle Enrichment

Reovirus virions and TC particles were enriched using a protocol that is standard in the field [44]. Briefly, L cells (2 × 10^8^) in suspension were adsorbed with media (mock-infected) or rsT1L or rsT3D^I^T1L1 reovirus at a multiplicity of ~10 plaque-forming units (PFU)/cell and incubated at 37 °C for 48 h. Virus-infected or mock-infected cells were pelleted by centrifugation at 3000 rpm for 10 min prior to resuspension in homogenization buffer (25 mM NaCl, 10 mM Tris-HCl, pH 7.4, 10 mM β-mercaptoethanol) and stored at −80 °C. Cell pellets were thawed, incubated with 0.14% deoxycholate for 30 min on ice, then sonicated in the presence of Vertrel XF to release virus particles from cells. Virions and TC particles, or mock preparations thereof, were separated by ultracentrifugation at 25,000 × *g* for 16 h in a 1.2–1.4 g/cm^3^ cesium chloride density gradient. Mock-virion and mock-TC preparations were collected by aligning a gradient containing virions and TC particles next to a gradient made using mock-infected L cells, marking the expected position of virions and TC particles on the mock gradient, aspirating liquid above the expected position, and transferring 250 µL of the gradient from the expected position of virions and TC particles into clean Eppendorf tubes. Mock preparations, complete virions, and TC particles were collected and dialyzed in virion storage buffer (150 mM NaCl, 15 mM MgCl_2_, 10 mM Tris-HCl, pH 7.4). When indicated, dialyzed particle preparations were rebanded by an additional round of ultracentrifugation at 25,000 × *g* for 16 h in a 1.2–1.4 g/cm^3^ cesium chloride density gradient prior to another round of dialysis.

### 2.4. Virus Particle Normalization

For experiments in which virions and TC particles were normalized by protein content, 10 µL of three independent stocks of virions and TC particles were resolved by SDS-10% PAGE and stained with colloidal Coomassie. Relative intensity of multiple reovirus protein bands was quantified with the Odyssey Infrared Imaging System (LI-COR, Lincoln, NE, USA) or ChemiDoc MP (BIO-RAD, Hercules, CA, USA). Virions and TC particles were subsequently resolved by SDS-10% PAGE, adjusting volumes to normalize relative intensity units, and stained with colloidal Coomassie. Then, relative intensity was quantified again to validate that protein content was successfully normalized for virion and TC samples. Volumes of virions and TC particles that provided equal relative intensity units were used to compare the infectivity and RNA content of equal particle numbers of virions and TC particles.

### 2.5. Bioanalyzer Analysis

Equivalent protein amounts (0.8–3 × 10^12^ particles) of rsT1L or rsT3D^I^T1L1 virions or TC particles were diluted in benzonase buffer (50 mM Tris-HCl, 2 mM MgCl_2_, pH 8.0) and either mock-treated or treated with 1 U/µL of benzonase (Millipore, Burlington, MA, USA) at room temperature or at 37 °C for 1 h to remove extra-particle nucleic acids. Based on protein normalization, virions were diluted approximately 3- to 4-fold relative to TC particles. Benzonase was inactivated with 0.5 M EDTA (pH 8.0), and RNA was extracted from virions and TC particles by TRIzol (Invitrogen, Waltham, MA, USA) extraction per manufacturer’s protocol. Concentration and quality of RNA were determined using a 2100 Bioanalyzer (Agilent, Santa Clara, CA, USA) and visualized as a gel display of electropherograms. Displays are automatically adjusted for fluorescence level so that RNA peaks were visible.

### 2.6. Library Preparation and Next-Generation RNA-Sequencing

Libraries were prepared for Illumina sequencing using RNA extracted from two or three independent preparations of purified, benzonase-treated rsT1L or rsT3D^I^T1L1 reovirus virions or TC particles, RNA extracted from benzonase-treated preparations of virion and TC preparations from mock-infected L cells (mock-virion and mock-TC controls), and from preparations of total RNA extracted from mock-infected or rsT1L-infected L cells in two independent experiments. To obtain total RNA preparations, L cell monolayers were adsorbed with media (mock-infected) or rsT1L reovirus at a multiplicity of 10 PFU/cell for 48 h. RNA was extracted from cells using TRIzol (Invitrogen) or from equivalent protein amounts of enriched reovirus particles (1–6 × 10^12^) using TRIzol LS Reagent (Invitrogen), according to the manufacturer’s protocol. Contaminating DNA was degraded by treating extracted RNA with RNase-free DNase I (New England Biolabs, Ipswich, MA, USA) for 10 min at 37 °C. RNA was re-extracted using TRIzol LS Reagent, and the concentration and quality of RNA was quantified using a 2100 Bioanalyzer (Agilent). RNA library preparation for Illumina sequencing was conducted using 5 ng of RNA and the NEBNext Ultra II RNA Library Prep Kit for Illumina (New England Biolabs), according to the manufacturer’s instructions. Briefly, ribosomal RNA was depleted from L cell samples via RNase H and DNase I digestion, and RNA was subsequently purified using RNAClean XP beads (Beckman Coulter, Brea, CA, USA). RNA was fragmented prior to first-strand and second-strand synthesis and RNAClean XP purification. PCR enrichment of adaptor ligated DNA was conducted using NEBNext Multiplex Oligos for Illumina (New England Biolabs) to produce Illumina-ready libraries. Illumina-ready libraries were sequenced by 150 base pair paired-end sequencing on the NovaSeq 6000 Sequencing System (Illumina, San Diego, CA, USA).

### 2.7. Sequence Analysis

Raw read quality was assessed using FastQC (v0.11.5) [45]. STAR (v2.7.3a) [46] was used to align reads to the *Mus musculus* genome, mm10, Genome Reference Consortium Mouse Build 38. Available online: http://hgdownload.soe.ucsc.edu/goldenPath/mm10/chromosomes/ (accessed on 6 April 2021) or to T1L and T3D reovirus segment sequences. GenBank Accession numbers for individual reference reovirus genome segments are M24734.1, AF378003.1, AF129820.1, AF461682.1, AF490617.1, AF174382.1, EF494445.1, L19774.1, M18389.1, M13139.1, EF494436.1, EF494437.1, EF494438.1, EF494439.1, EF494440.1, EF494441.1, EF494442.1, EF494443.1, and EF494444.1. Transcript quantification was done using featureCounts [47] using the paired-end mode to count reads that mapped uniquely. Then, the enriched transcripts were called using edgeR (v2.26.5) [48] with a Benjamini–Hochberg adjusted *p* value < 0.01. Only transcripts with counts per million (CPM) > 1 in at least two samples were included in the initial analysis. Enriched transcripts were further screened for at least an 8-fold change over matched mock preparations and an average log_2_CPM > 0.5 across samples of the particle type of interest. Comparisons of RNA content between samples or layers were conducted using edgeR (version 3.30.3). Pearson correlation coefficients were calculated using the log_2_ fold change as input to cor() function in R base package stats. ClusterProfiler (v3.12.0) [49] was used for the gene set overrepresentation analysis with GO terms (msigdbr_7.1.1) [50]. To illustrate Illumina reads mapping to the plus-strand and minus-strand of each viral genome segment, bam files were transformed into bedGraph files using bedtools (scaled to one million with bedtools use command “bedtools genomecov -bg -pc -scale 0.000001”) [51]. The bedGraph files were loaded into IGV to view the read distribution on target genes in a strand specific manner [52]. Figures were made using GraphPad Prism 8.4.3. Available online: http://www.graphpad.com (accessed on 6 April 2021).

### 2.8. RT-qPCR

RNA was isolated from the equivalent of 10^11^ particles of rsT1L virion and TC particle preparations using Trizol LS, per manufacturer’s protocol. Virion and TC RNA were primed with random hexamers (Invitrogen), and cDNA was generated by reverse transcription using SuperScript III reverse transcriptase (ThermoFisher, Waltham, MA, USA) per manufacturer’s protocol. Quantitative PCR amplification was performed using PowerUp SYBR Green Master Mix (ThermoFisher) and primers specific to the reovirus T1L S4 gene (F: 5′-CGCTTTTGAAGGTCGTGTATCA-3′; R: 5′-CTGGCTGTGCTGAGATTGTTTT-3′) or murine *HIST1H1E* (F: 5′-GGTACGATGTGGAGAAGAACAA-3′; R: 5′-CGCCTTCTTGTTGAGTTTGAAG -3′), *HIST1H2AI* (F: 5′-TCCGCAAAGGCAACTACTC -3′; R: 5′-TGATGCGCGTCTTCTTGT-3′), or *HIST2H3C2* (F: 5′- GATCGCGCAGGACTTCAA-3′; R: 5′-GGTTGGTGTCCTCGAACAG -3′).

### 2.9. Fluorescent Focus Assay

L cells (2  ×  10^4^ per well) were seeded into 96-well, black-walled plates and adsorbed with serial 10-fold dilutions of protein-normalized virion and TC preparations or volumes of serially diluted mock preparations at 37 °C for 1 h. After removing inocula, cells were washed and incubated in fresh medium at 37 °C for 24 h. After fixing with cold methanol, reovirus proteins in virus factories in the cell cytoplasm were detected using polyclonal reovirus antiserum in PBS containing 0.5% Triton X-100 at 37 °C, followed by washing and incubation with Alexa Fluor 488-labeled secondary IgG (Invitrogen) and DAPI (4′,6′-diamidino-2-phenylindole) to detect nuclei. Four fields of view per well were imaged with an ImageXpress Micro XL automated microscope (Molecular Devices, San Jose, CA, USA). Then, total and the percentage of infected cells were quantified with MetaXpress high-content image acquisition and analysis software (Molecular Devices).

### 2.10. Negative-Stain Electron Microscopy

Freshly glow-discharged Formvar/carbon grids (Electron Microscopy Services, Hatfield, PA, USA) were incubated with 2µL of purified reovirus virions or TC particles for one minute, washed twice by brief contact with a 50 µL water droplet, and stained for 10 s in 2% uranyl acetate. Imaging was performed on a Tecnai T12 operating at 100 kV using a drift-corrected AMT CMOS camera. Images were analyzed with FIJI ImageJ 1.53c [53].

### 2.11. Statistical Analysis

Statistical analyses were conducted using GraphPad Prism 8.4.3, www.graphpad.com. For FFA titers and RT-qPCR, results were found to be statistically different by one-way or two-way ANOVA. Then, titers of TC at each concentration were compared with those of virions or CT values were compared using Sidak’s multiple comparison test. Plaque titers of rsT1L virions and TC were compared by unpaired *t* test. The percentage of packaged viral reads for each reovirus segment for each particle type was compared with the percentage of total T1L reference genome length using a one-sample *t* test.

## 3. Results

### 3.1. Reovirus Top Component Particles Are Less Infectious Than Virions

**Figure 1:**
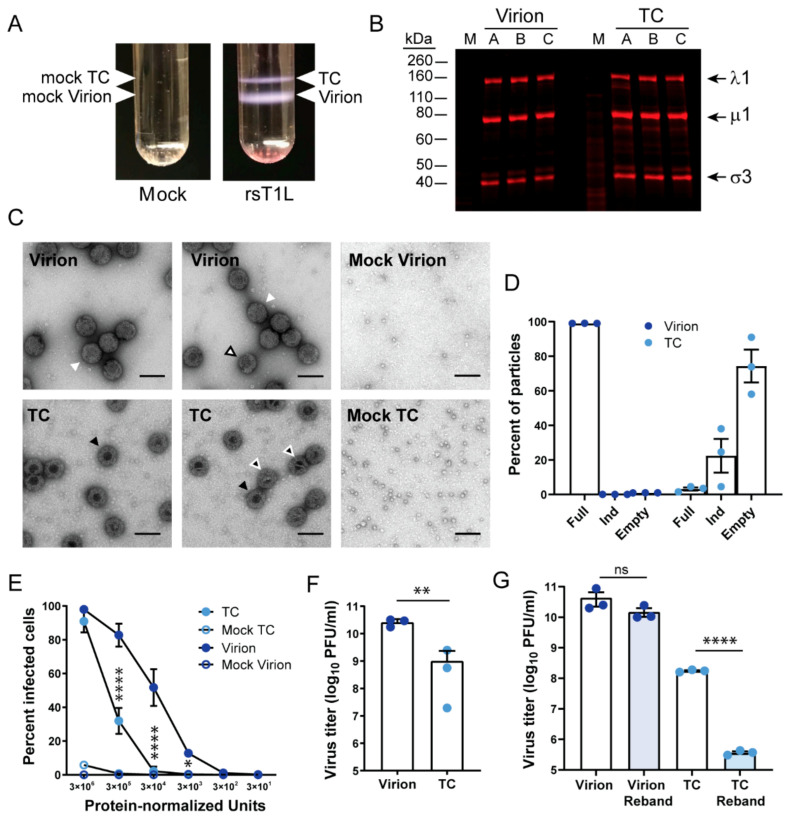
Low-density rsT1L reovirus TC particles have similar protein composition but are less infectious than virions. (**A**) L cells were adsorbed with medium (mock) or rsT1L reovirus at a MOI of 10 PFU/cell and incubated in suspension culture for 48 h prior to pelleting, sonication, and organic extraction. Ultracentrifugation in a cesium chloride density gradient was used to separate low-density TC particles from higher-density virions, as shown. Bands collected from the same positions in the gradient from processed mock-infected L cells are referred to as “mock virions” and “mock TC.” (**B**) Coomassie-stained SDS polyacrylamide gel on which equivalent protein concentrations of rsT1L reovirus virions and TC from three independently purified particle preparations (A-C) were resolved by electrophoresis and imaged. Presumptive major viral structural proteins are indicated. (**C**) Negative-stain electron micrographs of density gradient-purified virions, TC particles, mock virions, and mock TC particles. Scale bar = 100 nm. White arrowheads indicate “full” virions. A white arrowhead with black outline indicates a partially uncoated virion. Black arrowheads indicate “empty” TC particles. Black arrowheads with white outline indicate partially filled, indeterminate particles. (**D**) Quantitation of rsT1L virions and TC particles in EM images based on visual assessment as full, empty, or indeterminate. *n* = 3 independently purified preparations, at least 100 particles per preparation. (**E**) Monolayers of L cells were adsorbed with serial 10-fold dilutions of equivalent protein concentrations of rsT1L reovirus virions, TC particles, or mock preparations thereof for 1 h. Unbound particles were washed away, and cells were incubated for 16–20 h prior to fixation and staining to detect nuclei and reovirus proteins in virus factories. Percentage infected cells were detected in four fields of view and averaged. Error bars represent standard deviation. *n* = 3 independently purified preparations. *, *p* < 0.05; ****, *p* < 0.0001 for TC compared with virion by Sidak’s multiple comparison test. (**F**) Monolayers of L cells were adsorbed with equivalent protein concentrations of rsT1L reovirus virions, TC particles, or mock preparations thereof for 1 h. Cells were overlaid with a medium agar mixture and incubated for one week, with an intermittent feed, prior to staining to detect live cells. Error bars represent standard deviation. Plaque titers for individual samples are shown. *n* = 3 independently purified preparations. **, *p* < 0.01 compared with virion by unpaired *t* test. (**G**) Virions and TC particles enriched by cesium chloride gradient ultracentrifugation were collected and rebanded by cesium chloride gradient ultracentrifugation. Monolayers of L cells were adsorbed with equivalent protein concentrations of rsT1L reovirus virions and TC particles derived from initial enrichment or rebanding for 1 h. Cells were overlaid with a medium agar mixture and incubated for one week, with an intermittent feed, prior to staining to detect live cells. Error bars represent standard deviation. Plaque titers were determined in triplicate. ****, *p* < 0.0001 by unpaired *t* test.

Reovirus TC particles are reported to retain a low level of infectivity, despite ostensibly lacking viral genomic RNA [28]. To verify that TC particles are infectious, we enriched for recombinant strain (rs) T1L reovirus virions and TC particles by organic extraction and cesium chloride gradient ultracentrifugation from infected L cells. rsT1L TC could be cleanly separated from virions based on density (Figure 1A). We also processed mock-infected L cells using the same organic extraction and cesium chloride gradient ultracentrifugation approach and collected samples migrating at identical locations in the gradient as virions and TC particles (mock virions and mock TC). To compare the protein composition of TC particles and virions, we resolved the enriched particles by SDS-PAGE, stained the proteins with colloidal Coomassie, and quantified protein band intensity. Using this approach, we were able to normalize for protein content between virions and TC particles based on protein band intensity per volume of loaded sample. After normalizing, we found that the relative proportions of reovirus proteins were approximately equal for virions and TC particles, although a few additional proteins, including bands migrating slightly below λ1 and µ1, were detected more prominently in TC than virion preparations (Figure 1B). This normalization process was used in subsequent experiments to obtain equivalent amounts of virions and TC particles. By negative-stain electron microscopy (EM), enriched virions presented as electron-lucent particles, while most enriched TC particles had a dark, electron-dense interior, suggesting the absence of genomic RNA, as anticipated (Figure 1C) [26,28]. Some TC particles had a partially obscured or electron-lucent interior, though it was usually at least partially dark. Both virions and TC particles were about 80 nm in diameter. A noteworthy observation made using negative-stain EM was the detection of proteins that appeared as stacked ring-like structures. Far more of these structures were present in TC than virion preparations. However, they also were present at higher concentrations in mock-TC than mock-virion preparations, suggesting they are cellular protein complexes that migrate with a similar density to reovirus particles. To better assess the types of particles present, we quantified at least 100 particles in each of three independent preparations of rsT1L virions and TC particles based on appearance by negative-stain EM. We found that ~1% of particles in virion preparations appeared completely electron dense in the center, suggesting a low level of TC particle contamination (Figure 1D). About 3% of particles in TC preparations appeared completely electron lucent, suggesting low-level virion contamination. Additionally, 22% of particles in TC preparations were partially dark and partially lucent in the center; these “indeterminate” particles might have packaged some viral or host RNA but not a complete reovirus genome. These observations suggest that our gradient centrifugation and manual fractionation approach permitted strong enrichment but not absolute purification of T1L TC particles, which are similar in protein composition to virions but appear to lack all or most of the viral genome.

To quantify infectivity of the two enriched particle types, we adsorbed L cells with serial dilutions of protein-normalized rsT1L virions and TC particles and quantified infected cells after a single infectious cycle using a fluorescent focus assay. At the lowest dilution tested, both virions and TC particles could achieve high levels of infectivity (Figure 1E). However, with non-saturating concentrations of particles, virions were ~2.5 to 25 times more infectious than TC particles, and TC required ~100 times more particles than virions to infect at least 1% of cells. Titration of virions and TC particles by plaque assay indicated that virions contained an average of ~30 times more infectious PFU per protein-normalized unit than TC particles, though the range was broad, from ~10–1300 times more infectious units for the three independent preparations (Figure 1F). There was no gross visible difference in plaque size between virions and TC particles (not shown). To further assess the effects of contaminating virions on TC particle infectivity, we enriched a preparation of virions and TC particles with two sequential rounds of organic extraction and cesium chloride gradient ultracentrifugation. We collected and determined the titer of protein-normalized virion and TC particle samples following each round of enrichment (Figure 1G). In the initial enriched preparations, protein-normalized virions contained ~ 250 times as many infectious units per volume as TC particles. The titer of virions decreased slightly but not significantly per protein-normalized unit following rebanding. However, the titer of TC particles decreased to nearly 1/500th the infectivity of the initial enriched TC preparation per protein-normalized unit following rebanding. Together, these findings suggest that enriched virion preparations are far more infectious than TC particle preparations, but much of the residual infectivity detected in enriched TC particle preparations likely derives from low levels of contaminating virions.

### 3.2. Reovirus Particles Contain Viral Double-Stranded RNA

**Figure 2:**
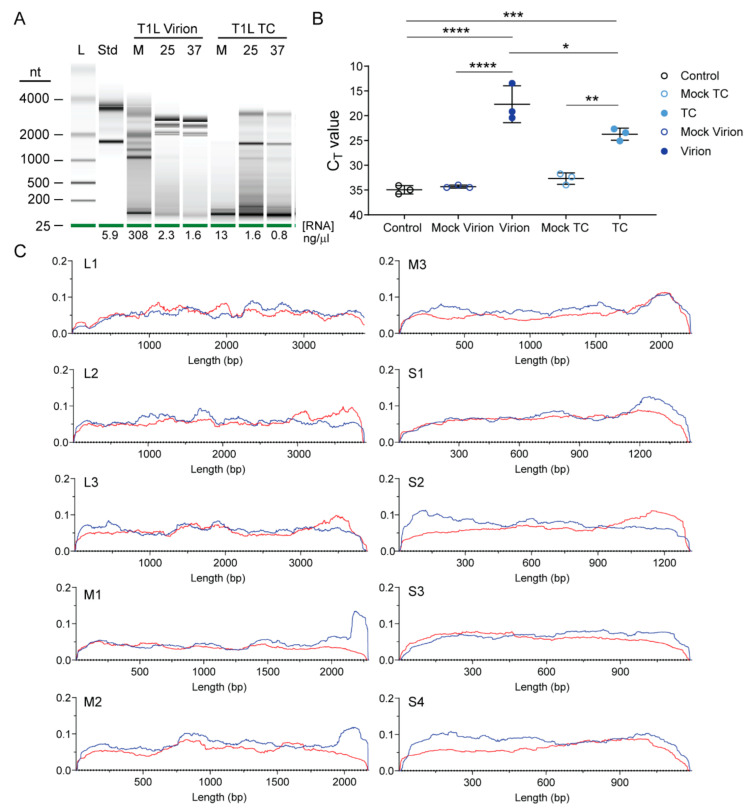
Enriched reovirus rsT1L TC particles package viral RNA. (**A**) Enriched rsT1L virions and TC particles were mock treated at 25 °C (M) or treated with benzonase at 25 °C (25) or 37 °C (37) to remove extra-particle nucleic acids. RNA was extracted, quantified, and resolved on a pico RNA chip using an Agilent Bioanalyzer. Shown are electrophoresis results, with the size of the ladder (L) in nucleotides indicated. A standard (Std) indicates eukaryotic 18s and 28s rRNA peaks. RNA concentration is indicated below each lane. (**B**) S4 RT-qPCR analysis of virions and TC particles. RNA was extracted from three independent, protein normalized rsT1L virion and TC particle preparations and equal volumes of a contemporaneously purified mock virion or TC preparation. cDNA was reverse transcribed using random hexamers, and qPCR reactions were conducted in the presence of primers specific for T1L S4. Nuclease-free water was added to control reactions in the place of template RNA. Shown are raw C_T_ values for the three independent rsT1L particle preparations or single mock preparations in triplicate. *, *p* < 0.05; **, *p* < 0.01; ***, *p* < 0.001; ****, *p* < 0.0001 by Sidak’s multiple comparison test. (**C**) Scaled Illumina read counts at each site for each segment in a representative rsT1L TC particle preparation. Segment identity and length (*x* axis) in bases are shown. Multiplication of the scaled *y* axis factor by 1,000,000 will reveal coverage at each site in CPM for plus-strand and minus-strand reads, which are indicated by red and blue lines, respectively.

To visualize the RNA content of enriched rsT1L virion and TC particle preparations based on electrophoretic mobility, we used Bioanalyzer. Particles were mock-treated or treated with benzonase to remove extra-particle nucleic acids. Then, RNA was extracted and resolved. RNA concentration and electrophoretic profiles differed markedly between mock-treated and benzonase-treated particles and between virions and TC particles. In the absence of benzonase treatment, strong signals from rsT1L virion-extracted RNA were detected at a small size between 25 and 200 nt and then from ~1000 to nearly 4000 nt, with distinct signals from ~1000 to 2000 nt, which may represent reovirus +RNAs (Figure 2A). Following benzonase treatment, RNA concentration was reduced to less than 1/100th the untreated level. Signal for the smallest RNAs largely disappeared, and RNA molecules packaged within rsT1L virions exhibited a distinct laddering pattern between ~2000 and 3000 nt, which may represent reovirus dsRNA genome segments. Overall RNA concentrations for protein-normalized TC particle equivalents were substantially lower than those of virions. In the absence of benzonase treatment, RNA extracted from TC particle preparations detectably contained only small RNAs between 25 and 200 nt. Following benzonase treatment of TC particles, RNA concentration was reduced to ~1/10th the untreated level. Small RNAs were still detected in TC-extracted RNA, as were many other bands, including two that were similar in size to 18s and 28s ribosomal RNA (rRNA). These findings suggest that TC particles encapsidate RNA, perhaps including small RNAs, but they do not encapsidate similar levels of viral genomic RNA as do virions.

To determine whether TC particles encapsidate viral RNA, we isolated RNA from protein-normalized equivalents of enriched rsT1L virions and TC particles or from equal volumes of contemporaneously purified mock preparations thereof. We generated cDNA by reverse transcription with random hexamers and quantified the relative abundance of S4 transcripts using primers specific for reovirus T1L S4 +RNA. We found that purified TC particles contain significantly more S4 +RNA than mock TC preparations (Figure 2B). However, consistent with Bioanalyzer results and their enhanced infectivity, virions contained significantly more S4 +RNA than TC particles (Figure 1D,E and Figure 2A,B).

**Figure 3:**
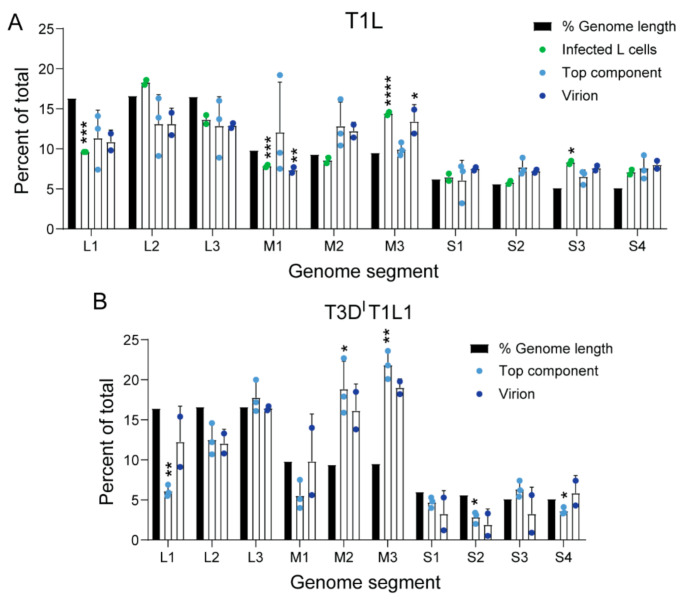
Percentage of packaged viral reads for each reovirus segment. The percentage of total T1L reference genome length was calculated for each genome segment and is indicated with a black bar. Numbers of viral reads from Illumina-ready libraries mapping to each segment were identified by alignment with the reference sequence. The percentage of total viral reads was calculated for each particle type. Shown are percentages calculated for TC (light blue), virions (dark blue), or infected L cells (green) for rsT1L (**A**) or rsT3D^I^T1L1 (**B**). Error bars represent standard deviation. *n* = 2 or 3 independent library preparations. *, *p* < 0.05; **, *p* < 0.01; ***, *p* < 0.001; ****, *p* < 0.0001 compared with percentage of total T1L reference genome length by one-sample *t* test.

To quantify and determine the strandedness of packaged rsT1L TC RNA compared with that of virions, we used NGS. To minimize the influence of extra-particle nucleic acids on sequencing results, we treated virions and TC particles with benzonase prior to RNA extraction. We generated randomly primed, directional libraries using RNA extracted from three independent preparations of rsT1L TC, two independent preparations each of rsT1L virions and total RNA from rsT1L-infected L cells, the cell type from which the particles had been purified, or from mock-virion and mock-TC preparations, and we sequenced them using Illumina technology. Both rsT1L virions and TC particles contained reads mapping to the full length of both strands for all ten T1L reovirus genome segments, although there were fewer viral reads in TC particles than in virions (Table 1). On average, rsT1L particles contained slightly more reads mapping to the plus-strand, 57.6% and 62.2% of total viral reads for virions and TC, respectively, than reads mapping to the minus-strand, 42.4% and 37.8% of total viral reads for virions and TC, respectively. These percentages are relatively consistent with the packaging of dsRNA, though slightly skewed towards +RNA. In contrast, for rsT1L-infected L cells, an average of 91% of reads mapped to the plus-strand, while 9% of reads mapped to the minus-strand, consistent with the presence of an abundance of +RNA transcripts in cells (Table 1). To determine if enriched TC particles package full-length segments, we analyzed read coverage for the plus- and minus-strands of all ten genome segments. For TC particles, read coverage was relatively uniform across the plus- and minus-strand of all genome segments, with the clearest exceptions in the minus-strands of the M1 and M2 genome segments, which exhibited denser coverage at the 5′ end (Figure 2C). Based on the assumption that each reovirus RNA segment should be represented equivalently, we adjusted the percentage of anticipated NGS reads based on segment length and determined whether the observed percentage of viral reads mapping to each segment matched our expectation (Figure 3A). For rsT1L-infected L cells, the observed percentage of reads mapping to L1 and M1 were lower and those mapping to M3 and S3 were higher than expected, suggesting that differences in +RNA stability or transcription efficiency may result in deviation from the anticipated RNA read ratios. However, only the percentage of reads mapping to M3 in rsT1L virions differed significantly from the segment’s percentage of total viral genome length, and no rsT1L TC segments differed in observed versus expected percentages of mapped reads based on segment length. Together, these findings suggest rsT1L TC particles encapsidate all ten reovirus dsRNA genome segments in the expected ratios for complete genomes but at reduced overall levels compared with virions. It is possible that some TC particles encapsidate incomplete reovirus genomes. However, considering the presence of a small percentage of “full” particles in TC preparations and the significant decrease in infectivity observed following TC rebanding, much of the detected viral RNA content may be derived from contaminating virions (Figure 1D,G). When using rsT1L virions as reference, rsT1L TC samples were positively correlated with the mock virion layer; the Pearson correlation coefficient was ~0.37, which is consistent with, but fails to definitively indicate, possible contamination between the layers.

### 3.3. Top Component Particles Contain Host RNA

**Figure 4:**
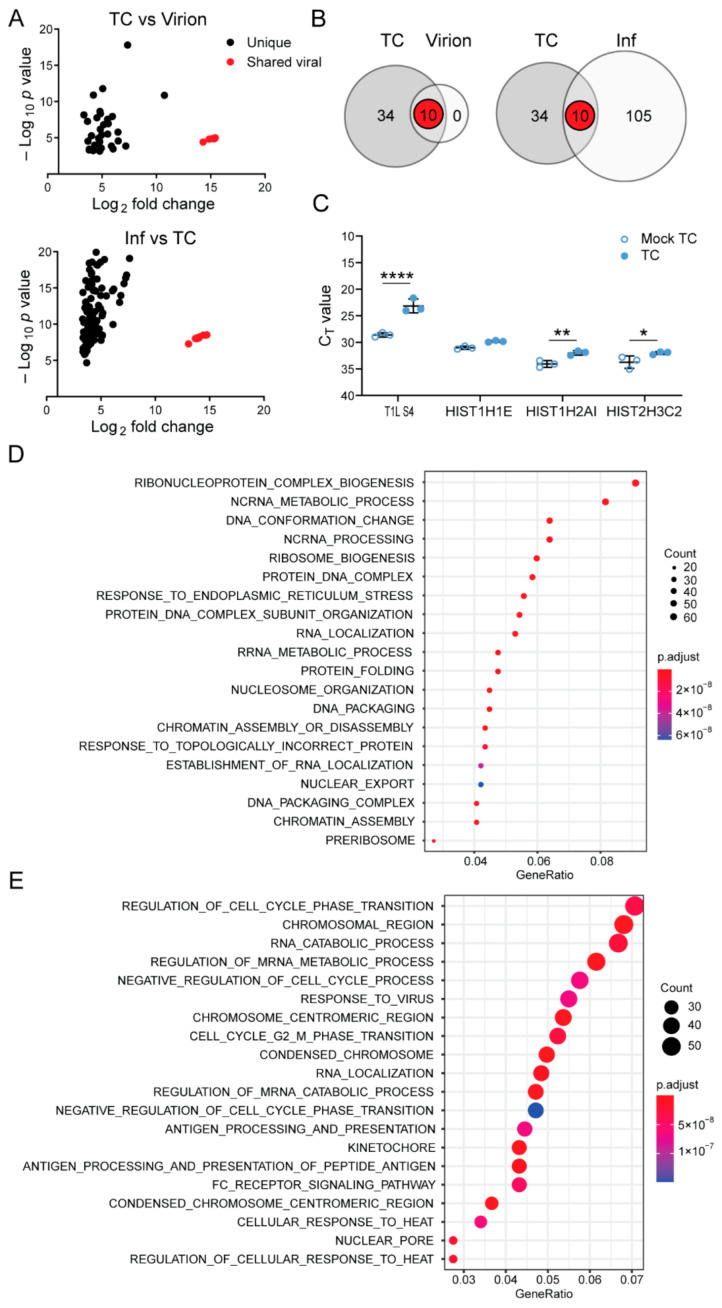
Reovirus rsT1L TC particles package host RNA. (**A**) Graphs showing host genes packaged by rsT1L TC that have a *p* value < 0.01 and fold change > 8 compared with mock TC. Red indicates shared viral genes between rsT1L TC and virions (upper) or rsT1L-infected L cells (lower). (**B**) Venn diagrams showing overlap in host genes with a *p* value < 0.01 and fold change > 8 between rsT1L TC and virions (left) or between rsT1L TC and rsT1L-infected L cells (right). Red indicates shared viral genes. (**C**) RT-qPCR validation of significant viral and host genes in TC compared with mock TC. RNA was extracted from three independent, protein-normalized rsT1L TC particle preparations and equal volumes of contemporaneously purified mock TC preparations. cDNA was reverse transcribed using random hexamers, and qPCR reactions were conducted in the presence of primers specific for the indicated target gene. Shown are raw C_T_ values. *, *p* < 0.05; **, *p* < 0.01; ****, *p* < 0.0001 by Sidak’s multiple comparison test. (**D**,**E**) Gene set overrepresentation analyses with GO terms. The *p* value is indicated by circle color, and transcript count is indicated by circle size, as shown in the legend. (**D**) Analysis of host genes packaged by rsT1L TC. (**E**) Analysis of host genes significantly upregulated in rsT1L-infected L cells.

NGS can identify non-viral RNA species, as well as viral RNA species, contained within virions and TC particles. While more than 99.9% of reads from RNA in rsT1L virions aligned with viral sequences, only ~18–74% of reads from RNA in rsT1L TC particles were viral, with remaining reads mapping to host transcripts (Table 2). To determine whether any cellular RNAs were preferentially packaged in rsT1L virions and TC particles, we compared read counts mapping to genes of the *Mus musculus* genome from virions and TC particles to mock particle preparations. We set stringent cutoffs of a *p* value < 0.01 and a greater than 8-fold change over mock to identify limited sets of host genes that were significantly enhanced in the data sets (Figure 4A and Table 3). All ten T1L reovirus genes were identified for both virions and TC and, unsurprisingly, exhibited the highest fold change over mock. rsT1L TC showed a significant increase in reads mapping to 34 host genes relative to mock TC preparations, while virions did not show a significant increase in read count for any host gene relative to mock virion preparations. Of note, about three-quarters of all genes that were significantly enriched for rsT1L TC were histone-encoding genes (Table 3). Many reads aligning with 18s rRNA were detected in TC preparations, in accordance with Bioanalyzer results, but they were not significant when compared with mock TC preparations (Figure 2A and Table 3). Gene set overrepresentation analyses indicated significant enrichment of host genes involved in ribonucleoprotein complex biogenesis, non-coding RNA processing, DNA conformation changes, and several other processes (Figure 4D). We used RT-qPCR to validate the presence of transcripts encoding host genes *HIST1H1E* and *HIST1H2AI*, which were significantly enriched by our standards, and *HIST2H3C2*, which was significant by *p* value but just missed our significance cutoff for CPM. Though differences were modest, as expected based on low numbers of mapped reads, two of these genes had significantly lower C_T_ values in TC than mock TC preparations, suggesting that they are enriched in rsT1L TC particles, and the third gene trended towards lower C_T_ values in TC particles (Figure 4C). These observations suggest that rsT1L virions specifically package viral transcripts to the exclusion of host transcripts, but numerous host transcripts are enriched in rsT1L TC particles.

It is possible that TC packaging of host RNAs is due to the abundance of RNA species within the cell and that increased expression of host genes in response to infection may drive non-specific packaging of host RNA. Therefore, we also conducted NGS analysis on total RNA extracted from mock-infected and rsT1L-infected L cells. Viral reads accounted for a significant portion (~76–80%) of total reads for RNA extracted from rsT1L-infected L cells. However, relative to mock-infected cells, rsT1L-infected cells displayed a significant increase in 105 host genes (Figure 4B). Of the 34 host genes significantly enhanced in rsT1L TC particles over mock TC particles, none were significantly increased in expression in rsT1L-infected cells compared with mock-infected cells (Figure 4A,B). Consistent with these findings, gene set overrepresentation analyses indicate distinct biological functions for genes upregulated in rsT1L TC and rsT1L-infected cells, with transcripts involved in cell-cycle regulation, the response to virus infection, RNA catabolic processes, and regulation of mRNA metabolic processes enriched in infected L cells (Figure 4E). Accordingly, the Pearson correlation coefficient between mock-virion versus mock-TC preparations compared with rsT1L virion versus TC preparations is ~0.007, which suggests that RNA associated with particles differs from host RNA in the mock corresponding layer. Together, these observations suggest that increased expression of host genes in response to infection is not the primary determinant of host gene packaging by rsT1L TC particles.

### 3.4. The Viral Polymerase Fails to Confer Complete Host RNA Packaging Specificity

Concurrent with or following encapsidation in assembling virus particles, viral +RNA transcripts associate with the RdRp λ3, which is encoded by the L1 segment and synthesizes minus-strand –RNA to form genomic dsRNA from +RNA templates [23,24]. Whether λ3 is important for viral RNA packaging is unknown. However, since rsT1L TC particles contain viral dsRNA, λ3 must associate with packaged viral +RNAs to synthesize the minus-strand. To determine if λ3 specifies the host genes packaged within TC particles, we sequenced RNA packaged by virions and TC particles of recombinant strain T3D^I^T1L1 reovirus. rsT3D^I^T1L1 is a T3D reovirus into which a T249I mutation has been engineered in the attachment protein that renders it resistant to proteolytic cleavage, and the λ3-encoding T3D L1 gene has been replaced with that of T1L [9]. rsT3D^I^T1L1 produced virions and TC particles in L cells. We generated libraries using RNA extracted from multiple preparations of enriched, benzonase-treated rsT3D^I^T1L1 virions and TC particles and sequenced them using Illumina technology. When using rsT3D^I^T1L1 virions as reference, rsT3D^I^T1L1 TC samples were positively correlated with the mock virion layer; the Pearson correlation coefficient was ~0.15, which fails to definitively indicate contamination between the layers. rsT3D^I^T1L1 TC particles contained reads mapping to all ten viral genome segments (Table 1 and Figure 3B). However, the percentages of viral reads mapping to each segment were less consistent with the expected percentages for rsT3D^I^T1L1 virions and TC particles than those of rsT1L. Whereas nearly all viral reads in rsT1L virions and TC particles mapped to segments in the expected percentages based on length, significantly more reads than expected mapped to the M2 and M3 segments, and significantly fewer reads mapped to the L1, L2, and S4 segments in rsT3D^I^T1L1 TC particles (Table 1 and Figure 3B). While they did not reach the level of statistical significance, similar trends were observed for rsT3D^I^T1L1 virions. Proportions of plus-strand to minus-strand viral reads for RNA extracted from rsT3D^I^T1L1 TC particles also differed substantially from the ~50% expected for genomic dsRNA (Table 1). Of all reads mapping to viral genes, on average, rsT3D^I^T1L1 virions had 65.2% plus-strand and 34.8% minus-strand reads, while rsT3D^I^T1L1 TC had 81.2% plus-strand and 18.8% minus-strand reads. These findings suggest that rsT3D^I^T1L1 TC particles package non-equimolar quantities of the ten reovirus genome segments and disproportionately package viral +RNA or fail to consistently synthesize the minus-strand.

**Figure 5:**
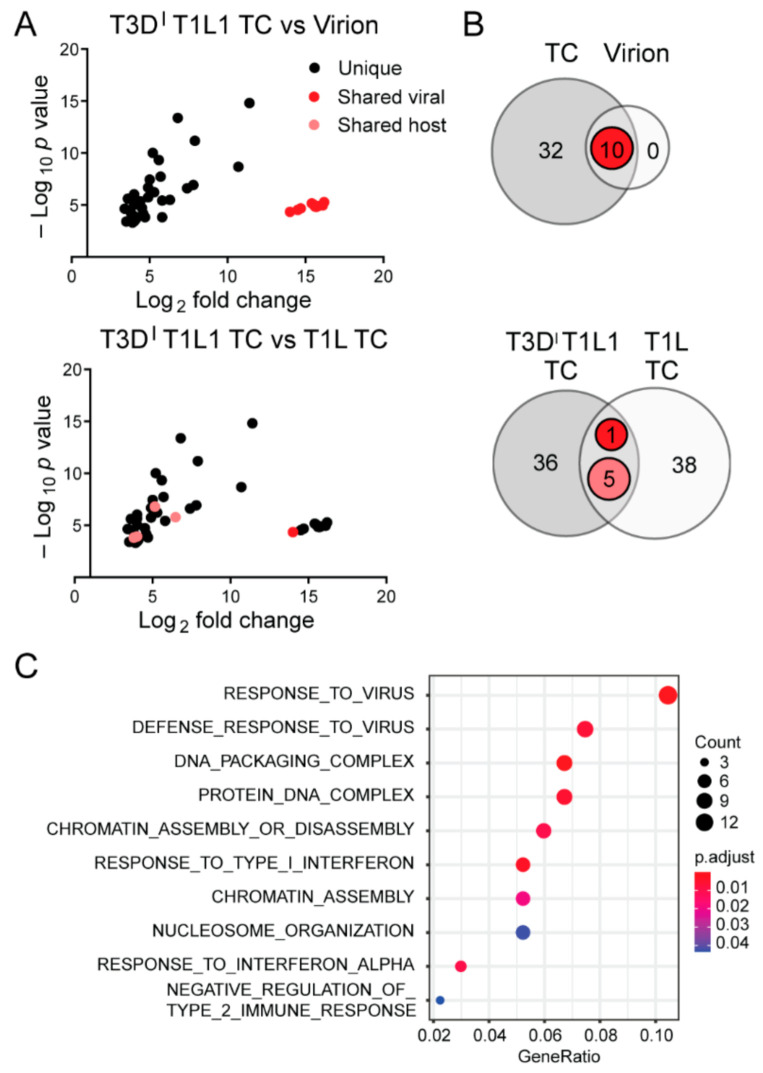
rsT1L TC particle host RNA packaging specificity is largely independent of the viral RdRp. (**A**) Graphs showing host genes packaged by rsT3D^I^T1L1 TC particles that have a *p* value < 0.01 and fold change > 8 compared with mock TC. Red and pink indicate shared viral and host genes, respectively, between rsT3D^I^T1L1 TC and virions (upper) or rsT1L TC (lower). (**B**) Venn diagrams showing overlap in host genes with a *p* value < 0.01 and fold change > 8 between rsT3D^I^T1L1 TC and virions (upper) or between rsT3D^I^T1L1 TC and rsT1L TC (lower). Red indicates shared viral genes, and pink indicates shared host genes. (**C**) Gene set overrepresentation analysis with GO terms for host genes packaged by rsT3D^I^T1L1 TC. The *p* value is indicated by circle color, and transcript count is indicated by circle size, as shown in the legend.

Similar to rsT1L virions, more than 99.8% of reads from RNA in rsT3D^I^T1L1 virions aligned with viral sequences (Table 2). However, consistently higher percentages of reads from RNA in rsT3D^I^T1L1 TC particles were viral (~66–76%), with remaining reads mapping to host transcripts. To determine whether any host cell RNAs were preferentially packaged within rsT3D^I^T1L1 virions and TC particles, we compared read counts mapping to genes of the *Mus musculus* genome from virions and TC particles to mock particle preparations using the same cutoff values applied in rsT1L analyses. The Pearson correlation coefficient between mock-virion versus mock-TC preparations compared with rsT3D^I^T1L1 virion versus TC preparations was approximately −0.008, which suggests that RNA associated with particles differs from host RNA in the mock corresponding layer. We identified 32 host RNAs that were significantly enriched in rsT3D^I^T1L1 TC particles relative to mock TC preparations (Table 3 and Figure 5A,B). No host genes were significantly enriched in rsT3D^I^T1L1 virions (Figure 5A,B). While five host genes that were enriched in rsT3D^I^T1L1 TC particles were also enriched in rsT1L TC particles, most significant host transcripts differed between the two groups (Figure 5A,B). Only one viral gene, L1, was shared between rsT1L and rsT3D^I^T1L1 TC. Histone-encoding genes comprised four of the five host genes that were upregulated in both rsT1L and rsT3D^I^T1L1 TC preparations (Table 3). The final shared gene was a predicted lincRNA. Gene ontology analysis suggests that rsT3D^I^T1L1 TC is more enriched in transcripts involved in interferon and host defense responses than rsT1L (Figure 5C and Table 3). Together, these findings suggest that there is overlap in the host transcripts packaged by rsT1L and rsT3D^I^T1L1 TC particles but that the viral RdRp is not primarily responsible for host transcript selection, which may differ among reovirus strains.

## 4. Discussion

In the current study, we used NGS to elucidate the content of fully infectious reovirus virions and low-density TC particles. Our data support the idea that packaging of the complete reovirus genome into virions is exquisitely specific. NGS analysis indicated that nearly all the RNA contained within cesium chloride gradient-purified reovirus virions is viral RNA. In fact, while TC preparations were significantly enriched in 32–34 host RNAs, no host transcripts were significantly enriched in virions based on our criteria (Figure 4A,B and Figure 5A,B; Table 3). Reads mapping to host RNA represented < 0.1% of total reads in enriched virions (Table 2). The proportion of reads corresponding to each of the ten viral genome segments in rsT1L virions was consistent with the expected proportion based on segment length, and encapsidated genome segments were largely double-stranded (Figure 2A and Table 1). Thus, reovirus virions rarely encapsidate host RNA.

Previous studies have found that despite appearing empty in electron micrographs, reovirus TC particles retain a level of infectivity much lower than that of virions [28]. Our analyses of the infectivity of virions and TC particles by plaque assay and fluorescent focus assay suggest a similar result (Figure 1E,F). However, virion contamination likely explains the majority of residual infectivity in our TC particle preparations, as much infectivity is lost upon rebanding (Figure 1G). Variation in virion contamination could contribute to the variability observed in the percentage of rsT1L TC reads that map to viral sequences (Table 2). While virion contamination likely accounts for a substantial proportion of viral reads and residual TC infectivity, it is possible that some viral RNA is packaged within TC particles. We harvested TC particles from the top of the gradient, and we observed only a small percentage of fully electron-lucent particles in TC preparations by negative-stain EM (Figure 1C,D). Some level of TC infectivity also may be accomplished through genetic complementation. If many TC particles package one or a small number of viral genome segments, then a complete set of viral +RNA transcripts could be provided when multiple particles are concurrently introduced into the same target cell and permit productive infection. In fluorescent-focus assays, near-saturating levels of infectivity were achieved for protein-normalized virions and TC particles when high particle numbers were used (Figure 1E). However, consistent with cooperative interactions among TC particles, 10-fold dilutions of inocula resulted in a much more rapid decrease in infectivity for TC particles than for virions. Thus, both virion contamination and genetic complementation may contribute to TC particle infectivity.

Although genetic complementation could in part explain TC infectivity, it is a poor fit with current packaging models. The observation that reovirus virions and TC particles form distinct bands in cesium chloride gradients and that each reovirus genome segment is packaged in rsT1L TC in the expected proportions based on segment length are consistent with “all-or-none” segment packaging (Figure 1A and Figure 3A). However, in negative-stain EM images, a significant percentage of TC particles contained partially filled centers, which could represent partially packaged viral genomes (Figure 1C,D). Based on observations for BTV, one might expect that small, viral RNA complex-nucleating segments would be overrepresented if only a few segments were being packaged within individual TC particles, but relatively increased levels of small segments were not detected in these particles (Figure 2C and Table 1). If TC infectivity is maintained through genetic complementation, reovirus RNA packaging may follow a less strict order based on size class than rotavirus and BTV, or RNA packaging may follow less strict guidelines in TC particles than in virions [36,37,38]. Future studies employing dilution and single-cell techniques may be useful in resolving these discordant observations.

It is unclear why TC particles fail to package a complete set of viral genome segments. Since read coverage for RNA packaged in rsT1L TC particles was relatively uniform across segment length, with approximately equal proportions of reads representing plus-strand and minus-strand RNA for nearly all segments, we do not anticipate that defective viral genomes contribute significantly to the failure of TC particles to package the complete viral genome (Figure 2C) [54]. Segments for which read coverage was substantially skewed were M1 and M2, which showed read enrichment localized to the 5′ end of the minus-strand. We hypothesize that these reads reflect abortive minus-strand synthesis, which is unlikely to influence packaging. It is possible that packaging of host transcripts by TC particles somehow precludes packaging of viral +RNA segments or complexes. If this is the case, it does not appear that TC particles become “filled” with host transcripts, based on particle density and appearance by negative-stain EM (Figure 1A,C). Therefore, some other mechanism must prevent complete viral RNA packaging into TC particles. Recent detection of collapsed, single-shelled particles in reovirus-infected cells suggests that the inner capsid may be assembled prior to being filled with RNA and RdRps [55]. However, TC particles appear to encapsidate the RdRp but not a complete viral genome. Thus, it is unclear whether these “star-like” single-shelled particles represent assembly intermediates or dead-end particle forms.

Viral and host RNA packaging by rsT1L and rsT3D^I^T1L1 virions and TC particles exhibited notable differences. While rsT1L virions and TC particles packaged reads mapping to most viral segments in proportion to length, in several instances, rsT3D^I^T1L1 TC particles deviated from expected proportions (Figure 3). Proportions of packaged plus-strand RNA in rsT3D^I^T1L1 TC particles, but typically not rsT1L TC particles, often were higher than expected for dsRNA (Table 1). Finally, rsT3D^I^T1L1 TC particles consistently packaged higher percentages of viral reads, relative to host reads, than rsT1L, though the level of virion contamination in rsT3D^I^T1L1 TC particle preparations was not quantified (Table 2). Future analyses of rsT3D^I^ may clarify whether differences in rsT1L and rsT3D^I^T1L1 viral RNA packaging are strain specific or result from mismatch with the RdRp. Of the 34 host transcripts significantly enriched in rsT1L TC particles, 5 were enriched in rsT3D^I^T1L1 TC particles, and rsT3D^I^T1L1 TC particles were enriched for another 27 distinct host transcripts (Figure 5A,B). Thus, the RdRp λ3 is not solely responsible for selecting host RNAs packaged in TC particles. Aside from λ3, several other reovirus proteins, including µNS, σ3, and σNS, interact with viral +RNA transcripts and potentially could contribute to overall RNA packaging specificity [22]. Polymerase co-factor µ2 interfaces with viral inclusions and the host cytoskeleton through interactions with polymerized microtubules [56,57,58]. Association with the host cytoskeleton dictates packaging efficiency and TC particle abundance for rsT1L and rsT3D reovirus [59]. Reovirus replication efficiency in Madin-Darby canine kidney cells can be modulated by the µ2- and λ3-encoding segments, with T3D exhibiting an apparent µ2-dependent packaging defect in these cells [60,61]. Thus, roles of µ2 and other viral proteins in host transcript packaging merit further exploration.

Our NGS analyses indicated that an average of 50% of reads for rsT1L TC particles and 71% of reads for rsT3D^I^T1L1 TC particles mapped to cellular RNA (Table 2). Of these, short, non-polyadenylated RNA species were enriched. Specifically, histone mRNAs, which are ~300–500 nucleotides in length and contain a conserved 3′ stem loop, represented the majority of cellular RNAs packaged by rsT1L TC particles and most of the shared genes packaged by both rsT1L and rsT3D^I^T1L1 TC particles [62]. Since reovirus +RNAs are non-polyadenylated and contain predicted stem-loop structures in terminal UTRs, and genome segment termini are critical for packaging, packaging may be preferential for transcripts that conserve these features [9,15,18,20,63,64]. In addition to highly structured, non-polyadenylated cellular RNAs, polyadenylated host transcripts were packaged within TC particles, particularly in rsT3D^I^T1L1 TC particles (Table 3). For rsT1L, there was no overlap in packaged transcripts and those that were upregulated in response to infection, and gene set overrepresentation analyses identified several distinct categories of RNAs enriched in rsT1L TC particles and rsT1L-infected L cells (Figure 4). These findings suggest that reovirus packaging of host RNA is facilitated through conserved RNA features rather than transcript abundance.

Whether TC packaging of host RNA has significant functional consequences for reovirus replication is an open question. Reovirus packages viral +RNA and uses it as a template for minus-strand synthesis to make the dsRNA genome [23]. Since TC particles package the viral RdRp and cellular RNA transcripts, these particles could conceivably generate dsRNA and synthesize nascent mRNA transcripts from cellular RNA (Figure 4 and Figure 5) [27]. Host RNA packaged within TC particles, however, was nearly always single-stranded, suggesting reovirus is incapable of using cellular RNAs as templates for replication (not shown). Furthermore, there were far fewer reads detected for most significantly enriched host RNAs than for viral +RNAs packaged by TC particles, even though total numbers of host transcript reads were high (Table 2). Rather than altering target cell biology, host RNA packaging may simply alter TC particle encapsidation of viral RNA or be permitted when a complete set of viral RNAs fails to be encapsidated.

RNA packaging by viruses belonging to the *Reoviridae* family is mediated by *cis*- and *trans*-segment interactions reliant upon specific nucleotide sequence and structural motifs. Here, we demonstrate that reovirus TC particles can package diverse cellular RNA transcripts, while virions fail to do so, supporting a highly selective genome packaging model for virions. Packaging of host transcripts within TC particles is not based solely on transcript abundance and may differ based on virus strain, suggesting some selectivity, but is not determined solely by the viral RdRp. We speculate that encapsidation of host transcripts is unlikely to significantly affect the biology of cells into which TC particles enter, as packaged cellular transcript abundance is low, and there is no apparent mechanism for host transcript exit or amplification. Rather, host transcript packaging may interfere with viral +RNA packaging or simply be permitted when a full complement of viral +RNAs fails to be packaged. Future studies are required to reveal the mechanism and outcome of host transcript packaging by reovirus TC particles.

## Figures and Tables

**Table 1:** Viral reads from rsT1L-infected L cells and rsT1L or rsT3D^I^T1L1 TC or virions.

Segment	Number and Percentage of Total Viral Reads									
	Inf-A ^a^	Inf-B	%	T1L TC-A	T1L TC-B	T1L TC-C	%	T1L V-A ^b^	T1L V-B	%
T1L L1	1,038,877	1,063,552	10	1,129,755	177,576	1,423,811	11	3,447,733	2,877,184	11
T1L L2	1,940,530	2,058,662	18	1,259,866	217,047	1,638,263	13	4,181,427	3,428,793	13
T1L L3	1,410,996	1,569,759	14	1,243,118	211,302	1,611,815	13	3,809,943	3,704,721	13
T1L M1	828,734	888,345	8	860,795	457,747	754,088	12	2,229,966	2,057,585	7
T1L M2	880,271	987,720	9	1,079,924	386,976	1,047,832	13	3,286,855	3,834,136	12
T1L M3	1,580,719	1,574,052	14	829,202	257,314	990,199	10	3,435,740	4,379,129	13
T1L S1	742,227	659,561	6	705,967	76,568	722,037	6	2,210,645	2,136,583	8
T1L S2	650,896	621,659	6	663,153	211,496	695,395	8	2,020,455	2,187,448	7
T1L S3	922,919	900,962	8	622,472	169,088	554,903	7	2,109,575	2,326,564	8
T1L S4	799,830	752,994	7	663,235	220,084	638,508	8	2,158,986	2,495,732	8
				T3D^I^ TC-A	T3D^I^ TC-B	T3D^I^ TC-C	%	T3D^I^ V-A	T3D^I^ V-B	%
T1L L1				765,380	755,871	841,398	6	4,508,769	2,323,188	12
T3D L2				1,607,504	1,377,489	1,876,766	13	3,165,210	3,399,842	12
T3D L3				1,902,672	2,076,988	3,083,154	18	4,751,091	4,271,775	17
T3D M1				825,554	652,109	620,491	6	4,088,339	1,424,936	10
T3D M2				1,756,670	2,928,330	2,766,220	19	4,041,153	4,738,918	16
T3D M3				2,218,413	2,812,836	3,649,313	22	5,792,592	4,661,471	19
T3D S1				526,488	684,413	619,586	5	347,908	1,347,464	3
T3D S2				378,205	403,546	313,405	3	137,275	835,851	2
T3D S3				685,219	691,058	1,147,250	6	261,883	1,439,514	3
T3D S4				378,875	527,151	514,224	4	2,156,059	1,105,882	6
Segment	Number and Percentage of Plus-Strand Viral Reads Per Segment ^c^									
	Inf-A	Inf-B	%	T1L TC-A	T1L TC-B	T1L TC-C	%	T1L V-A	T1L V-B	%
T1L L1	967,418	926,730	90	559,481	72,257	716,916	47	1,478,406	1,239,329	43
T1L L2	1,715,514	1,682,724	85	600,385	76,108	831,884	45	1,778,263	1,542,290	44
T1L L3	1,318,548	1,387,044	91	643,641	136,574	824,859	56	1,947,084	2,259,676	56
T1L M1	776,872	756,578	90	592,363	407,332	451,155	73	1,247,124	1,246,707	58
T1L M2	842,077	877,752	92	676,676	357,046	610,169	71	2,086,040	2,948,204	70
T1L M3	1,524,971	1,463,682	95	499,057	214,596	550,533	66	2,069,244	3,280,321	68
T1L S1	642,149	543,860	85	385,850	48,236	392,044	57	1,144,399	1,105,627	52
T1L S2	631,366	568,782	94	413,158	190,920	379,510	69	1,099,334	1,249,891	56
T1L S3	884,705	829,931	94	371,082	146,717	299,734	67	1,238,926	1,588,222	64
T1L S4	765,558	705,075	95	411,370	206,932	369,635	71	1,331,638	1,754,691	66
				T3D^I^ TC-A	T3D^I^ TC-B	T3D^I^ TC-C	%	T3D^I^ V-A	T3D^I^ V-B	%
T1L L1				420,930	444,649	462,292	56	2,284,404	1,284,049	53
T3D L2				1,176,817	1,101,902	1,632,328	80	1,614,887	2,418,628	61
T3D L3				1,625,208	1,809,948	2,809,072	88	2,431,490	3,306,435	64
T3D M1				496,598	436,268	424,148	65	2,036,735	878,312	56
T3D M2				1,600,316	2,775,413	2,623,817	93	2,195,291	4,406,569	74
T3D M3				2,035,177	2,626,693	3,547,829	94	3,120,135	4,304,327	73
T3D S1				421,810	604,230	561,397	86	198,146	1,167,135	72
T3D S2				257,660	299,371	250,122	74	71,453	620,844	63
T3D S3				617,938	619,140	1,122,183	93	134,518	1,292,697	71
T3D S4				296,978	417,187	449,194	82	1,113,725	889,520	66

^a^ Inf, total RNA from infected cells; A, B, and C refer to independent particle, RNA, and library preparations. ^b^ V, virions ^c^ Remaining viral reads totaling 100% and the number from the top portion of the table are minus-strand reads.

**Table 2:** Percentage of viral and cellular reads from rsT1L-infected L cells and rsT1L or rsT3D^I^T1L1 TC particles or virions.

Sample	Viral CPM	Host CPM	Percent Viral	Percent Host
Mock Inf-A ^a^	413	999,587	<0.1	>99.9
Mock Inf-B	332	999,668	<0.1	>99.9
T1L Inf-A	762,352	237,648	76	24
T1L Inf-B	798,981	201,019	80	20
Mock TC	387	999,613	<0.1	>99.9
T1L TC-A	587,065	412,935	59	41
T1L TC-B	178,652	821,348	18	82
T1L TC-C	742,350	257,650	74	26
T3D^I^T1L1 TC-A	663,806	336,194	66	34
T3D^I^T1L1 TC-B	700,570	299,430	70	30
T3D^I^T1L1 TC-C	763,056	236,944	76	24
Mock Virion	697	999,303	<0.1	>99.9
T1L Virion-A	999,792	208	>99.9	<0.1
T1L Virion-B	999,609	391	>99.9	<0.1
T3D^I^T1L1 Virion-A	999,901	99	>99.9	<0.1
T3D^I^T1L1 Virion-B	998,510	1490	99.9	0.1

^a^ Inf, total RNA from mock-infected or T1L reovirus-infected cells; A, B, and C refer to independent sample and library preparations.

**Table 3:** Viral and host transcripts packaged by rsT1L and rsT3D^I^T1L1 particles.

Gene Identifier	Log_2_FC ^a^	*p* Value	SYMBOL
rsT1L Virions Significant Genes ^b^			
gb|M24734.1|	16.8	7.3 × 10^−6^	T1L L1
gb|AF378003.1|	17.4	3.4 × 10^−6^	T1L L2
gb|AF129820.1|	17.2	3.7 × 10^−6^	T1L L3
gb|AF461682.1|	17.8	2.9 × 10^−6^	T1L M1
gb|AF490617.1|	17.7	2.5 × 10^−6^	T1L M2
gb|AF174382.1|	17.8	2.5 × 10^−6^	T1L M3
gb|EF494445.1|	17.5	2.4 × 10^−6^	T1L S1
gb|L19774.1|	17.6	2.3 × 10^−6^	T1L S2
gb|M18389.1|	17.6	2.3 × 10^−6^	T1L S3
gb|M13139.1|	17.5	2.4 × 10^−6^	T1L S4
rsT1L TC Significant Genes			
gb|M24734.1|	14.3	3.9 × 10^−5^	T1L L1
gb|AF378003.1|	15.3	1.4 × 10^−5^	T1L L2
gb|AF129820.1|	15.3	1.4 × 10^−5^	T1L L3
gb|AF461682.1|	15.4	1.1 × 10^−5^	T1L M1
gb|AF490617.1|	15.4	1.3 × 10^−5^	T1L M2
gb|AF174382.1|	15.3	1.4 × 10^−5^	T1L M3
gb|EF494445.1|	15.2	1.2 × 10^−5^	T1L S1
gb|L19774.1|	15.4	1.0 × 10^−5^	T1L S2
gb|M18389.1|	14.9	1.6 × 10^−5^	T1L S3
gb|M13139.1|	15.1	1.3 × 10^−5^	T1L S4
ENSMUST00000083211.1	7.2	1.4 × 10^−4^	Vaultrc5
ENSMUST00000062045.3	6.0	3.5 × 10^−4^	Hist1h1e
ENSMUST00000147537.5	6.5	2.8 × 10^−5^	Lmna
ENSMUST00000098843.2	5.9	1.4 × 10^−4^	Hist2h3b
ENSMUST00000079251.7	4.9	2.8 × 10^−4^	Hist1h2bg
ENSMUST00000102967.2	4.3	4.1 × 10^−4^	Hist1h4c
ENSMUST00000074752.3	4.2	5.9 × 10^−4^	Hist1h2ak
ENSMUST00000045301.8	4.8	6.9 × 10^−4^	Hist1h1d
ENSMUST00000099703.4	4.3	4.5 × 10^−4^	Hist1h2bb
ENSMUST00000102979.1	3.8	5.0 × 10^−4^	Hist1h4n
ENSMUST00000102983.1	4.3	1.1 × 10^−4^	Hist1h4k
ENSMUST00000070124.4	5.5	3.2 × 10^−6^	Hist1h2ai
ENSMUST00000102969.5	4.8	3.7 × 10^−5^	Hist1h2ae
ENSMUST00000091752.4	4.8	2.2 × 10^−5^	Hist1h3c
ENSMUST00000087714.5	4.7	1.1 × 10^−5^	Hist1h4j
ENSMUST00000078369.2	5.0	4.1 × 10^−6^	Hist1h2ab
ENSMUST00000091709.2	6.0	1.2 × 10^−8^	Hist1h2bn
ENSMUST00000144964.7	4.9	4.7 × 10^−9^	Pex6
ENSMUST00000171127.3	3.7	2.9 × 10^−5^	Hist1h2ac
ENSMUST00000091703.2	5.6	1.1 × 10^−7^	Hist1h3b
ENSMUST00000091708.5	3.8	3.7 × 10^−4^	Hist1h2al
ENSMUST00000105106.1	4.8	6.5 × 10^−7^	Hist1h2bf
ENSMUST00000188775.1	4.0	5.3 × 10^−6^	Hist1h3h
ENSMUST00000091756.1	5.5	2.9 × 10^−8^	Hist1h2bl
ENSMUST00000224651.1	5.2	1.6 × 10^−7^	Hist1h2bm
ENSMUST00000224359.1	4.5	1.8 × 10^−8^	Hist1h2bh
ENSMUST00000136269.7	4.2	1.3 × 10^−11^	Rpl7a
ENSMUST00000149925.7	5.1	1.6 × 10^−12^	Ctu2
ENSMUST00000073261.2	10.8	1.4 × 10^−11^	Hist1h2af
ENSMUST00000090776.6	4.8	2.3 × 10^−9^	Hist1h2ad
ENSMUST00000181242.1	6.5	1.7 × 10^−6^	Gm26870 lincRNA ^c^
ENSMUST00000159697.1	7.4	1.6 × 10^−18^	Acat2
ENSMUST00000107249.7	3.3	6.9 × 10^−9^	Rpl27
ENSMUST00000091751.2	3.7	5.6 × 10^−8^	Hist1h2an
rsT3D^I^-T1L1 Virions Significant Genes			
gb|M24734.1|	18.1	2.9 × 10^−6^	T1L L1
gb|EF494436.1|	17.3	4.2 × 10^−6^	T3D L2
gb|EF494437.1|	17.6	3.9 × 10^−6^	T3D L3
gb|EF494438.1|	17.9	2.7 × 10^−6^	T3D M1
gb|EF494439.1|	18.1	2.8 × 10^−6^	T3D M2
gb|EF494440.1|	18.1	3.1 × 10^−6^	T3D M3
gb|EF494441.1|	16.6	4.2 × 10^−6^	T3D S1
gb|EF494442.1|	15.7	6.3 × 10^−6^	T3D S2
gb|EF494443.1|	16.7	4.0 × 10^−6^	T3D S3
gb|EF494444.1|	17.7	2.6 × 10^−6^	T3D S4
rsT3D^I^-T1L1 TC Significant Genes			
gb|M24734.1|	14.0	4.6 × 10^−5^	T1L L1
gb|EF494436.1|	15.6	1.4 × 10^−5^	T3D L2
gb|EF494437.1|	15.8	1.3 × 10^−5^	T3D L3
gb|EF494438.1|	14.5	3.0 × 10^−5^	T3D M1
gb|EF494439.1|	16.1	1.1 × 10^−5^	T3D M2
gb|EF494440.1|	15.7	1.5 × 10^−5^	T3D M3
gb|EF494441.1|	15.5	8.7 × 10^−6^	T3D S1
gb|EF494442.1|	15.4	7.1 × 10^−6^	T3D S2
gb|EF494443.1|	16.2	5.2 × 10^−6^	T3D S3
gb|EF494444.1|	14.7	2.1 × 10^−5^	T3D S4
ENSMUST00000098843.2	5.8	1.5 × 10^−4^	Hist2h3b
ENSMUST00000045540.3	7.8	1.2 × 10^−7^	Socs7
ENSMUST00000033930.4	4.7	1.5 × 10^−4^	Dusp4
ENSMUST00000032094.6	7.4	2.5 × 10^−7^	Fbxl14
ENSMUST00000046929.6	4.6	7.1 × 10^−5^	Usp31
ENSMUST00000147545.7	5.8	3.7 × 10^−6^	Ccdc6
ENSMUST00000079869.12	3.5	4.0 × 10^−4^	Znrf2
ENSMUST00000050063.8	4.5	1.9 × 10^−5^	Arf6
ENSMUST00000178344.2	4.1	2.6 × 10^−4^	Itpripl2
ENSMUST00000052838.10	5.3	5.9 × 10^−7^	Mib1
ENSMUST00000007980.6	4.0	3.7 × 10^−4^	Hnrnpa0
ENSMUST00000093962.4	3.9	4.9 × 10^−4^	Ccnd1
ENSMUST00000106113.1	3.8	5.3 × 10^−5^	Foxk2
ENSMUST00000073109.11	6.8	4.2 × 10^−14^	Ctdspl
ENSMUST00000058550.14	5.7	1.9 × 10^−8^	Ccni
ENSMUST00000035220.11	4.9	2.1 × 10^−7^	Prkar2a
ENSMUST00000022875.6	4.9	1.8 × 10^−6^	Ank
ENSMUST00000044954.6	3.4	2.3 × 10^−5^	Slc30a1
ENSMUST00000069180.7	5.6	4.8 × 10^−10^	Zcchc24
ENSMUST00000102824.3	11.4	1.6 × 10^−15^	Ifit1
ENSMUST00000085425.4	7.9	6.8 × 10^−12^	Isg15
ENSMUST00000070124.4	4.2	1.6 × 10^−4^	Hist1h2ai
ENSMUST00000149978.1	5.0	3.6 × 10^−8^	Inafm2
ENSMUST00000050467.8	4.0	9.4 × 10^−7^	Tob2
ENSMUST00000013807.7	3.6	2.5 × 10^−6^	Pten
ENSMUST00000102825.3	10.7	2.1 × 10^−9^	Ifit3
ENSMUST00000078369.2	4.0	1.2 × 10^−4^	Hist1h2ab
ENSMUST00000181242.1	6.3	3.2 × 10^−6^	Gm26870 lincRNA
ENSMUST00000008537.9	4.0	2.2 × 10^−6^	Carhsp1
ENSMUST00000034832.7	5.2	1.0 × 10^−10^	Ptpn9
ENSMUST00000028648.2	4.0	9.5 × 10^−6^	Syt13
ENSMUST00000224651.1	4.4	4.4 × 10^−6^	Hist1h2bm

^a^ FC, fold change over mock; ^b^ Significant genes exhibit at least 8-fold change over matched mock preparations and *p* values < 0.01. ^c^ Symbols in gray text initially showed as “NA” but were identified using a manual search at ensembl.org.

## Data Availability

Data generated from Illumina RNA-seq can be accessed at NCBI Gene Expression Omnibus (https://www.ncbi.nlm.nih.gov/geo/) under accession number GSE164270. Illumina RNA-seq data for rsT1L virions can also be accessed at NCBI’s Sequence Read Archive (https://www.ncbi.nlm.nih.gov/sra) under BioProject accession PRJNA669717.
